# A case of paroxysmal cold hemoglobinuria complicated by latent syphilis

**DOI:** 10.1093/omcr/omae009

**Published:** 2024-03-25

**Authors:** Tsuyoshi Hirata, Naoko Kubota, Kazuaki Fukushima, Erika Takami, Tsuyoshi Kato, Tomomi Okamoto

**Affiliations:** Department of Internal Medicine, Tokyo Metropolitan Cancer and Infectious Diseases Center Komagome Hospital, Tokyo, Japan; Department of Internal Medicine, Tokyo Metropolitan Cancer and Infectious Diseases Center Komagome Hospital, Tokyo, Japan; Department of Infectious Diseases, Tokyo Metropolitan Cancer and Infectious Diseases Center Komagome Hospital, Tokyo, Japan; Department of Internal Medicine, Tokyo Metropolitan Cancer and Infectious Diseases Center Komagome Hospital, Tokyo, Japan; Department of Internal Medicine, Tokyo Metropolitan Cancer and Infectious Diseases Center Komagome Hospital, Tokyo, Japan; Department of Internal Medicine, Tokyo Metropolitan Cancer and Infectious Diseases Center Komagome Hospital, Tokyo, Japan

## Abstract

An 80-year-old man presented in December with the main complaint of jaundice. Blood tests revealed hemolytic anemia and renal dysfunction. Positive syphilis serology results led to a diagnosis of untreated latent syphilis. A positive direct Coombs test led to a diagnosis of autoimmune hemolytic anemia (AIHA). Antibiotics were started for the syphilis, with improvement in the anemia and renal dysfunction observed. However, paroxysmal intravascular hemolysis occurred after his discharge. Based on a positive Donath-Landsteiner (D-L) test, paroxysmal cold hemoglobinuria (PCH) diagnosis was made. The hemolytic anemia improved after further treatment for syphilis, and further avoiding exposure to cold.

## INTRODUCTION

Paroxysmal cold hemoglobinuria (PCH) is a very rare type of autoimmune hemolytic anemia (AIHA). PCH is classified as a condition that is associated with cold, accounting for only 1% of all AIHAs [1]. PCH is seen in young children after viral illnesses, in some hematologic malignancies, and in syphilis cases. In the past, there were many syphilitic cases reported; however, at the present time, most reported cases are non-syphilitic. We report a case of acute paroxysmal cold hemoglobinuria in an elderly male with latent syphilis.

## CASE REPORT

**Figure 1:**
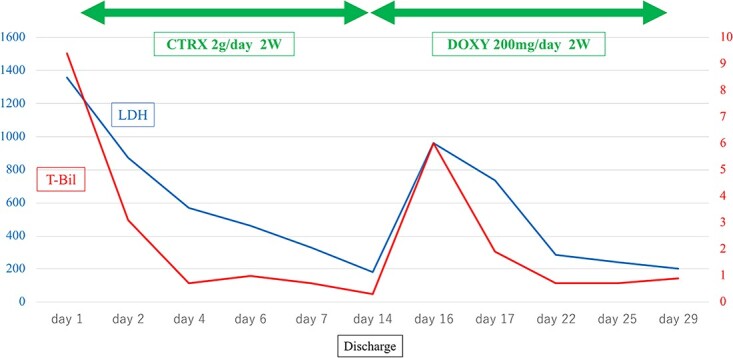
Time course of total bilirubin (T-bil) and lactic acid dehydrogenase (LDH) values.

An 80-year-old man with a history of hypertension, chronic heart failure, and dyslipidemia presented to our hospital in December with the main complaint of jaundice. Laboratory studies revealed anemia with hemolysis, an elevated LDH level (LDH 1358 U/l), and indirect hyperbilirubinemia (total bilirubin: 9.4 mg/dl, direct bilirubin: 0.7 mg/dl). Syphilis serology was found to have positive qualitative RPR (389.9×) and TPLA (9937.0×). Based on these findings, we diagnosed the patient with untreated latent syphilis. Blood tests showed elevated reticulocytes, decreased serum haptoglobin, and positive IgG and C3b3d in a direct Coombs test. After reviewing his lab results, a diagnosis of AIHA was made. Bone marrow examination showed no findings suggestive of malignancy. After admission, antibiotics (ceftriaxone 2 g/day) were started for the syphilis for 14 days, with a subsequent improvement in the anemia, renal dysfunction, LDH level, and indirect hyperbilirubinemia observed. The baseline (Day 1: at admission) and follow-up (Day 14: at the day before discharge) laboratory studies are shown in Table. On day 15, the patient was discharged to home. However, nausea and vomiting appeared in the morning after discharge, along with a flare-up of his jaundice, thereby resulting in re-hospitalization on day 16 (Fig. 1).

Blood tests after the re-hospitalization showed recurrent hemolytic anemia (Table 1). Because of these repeated episodes of paroxysmal hemolytic anemia, along with his history of living without a heater in his home during the winter, cold AIHA was suspected. As a result, the patient underwent cold agglutination and D-L tests.

**Table 1:** Laboratory Data on Day 1, Day 14, and Day 16. Day 1: Hospitalization, Day 14: The day before discharge, Day 16: Re-hospitalization

	Day 1	Day 14	Day 16	
WBC	10.7	3.7	9.8	×10^3^/μl
Hb	10.3	8.9	8.3	g/dl
PLT	26.2	36.1	31.1	×10^4^/μl
HCT	30.6	27	23.1	%
MCV	95	93	97	fl
T-Bil	9.4	0.3	6	mg/dl
D-Bil	0.7	0.1	0.5	mg/dl
AST	65	30	71	U/l
ALT	16	26	24	U/l
LDH	1358	181	963	U/l
ALP	327	264	306	U/l
Cr	3.2	1.27	1.61	mg/dl
UN	77	13	39	mg/dl
CRP	4.51	2.5	2.68	mg/dl

**Figure 2:**
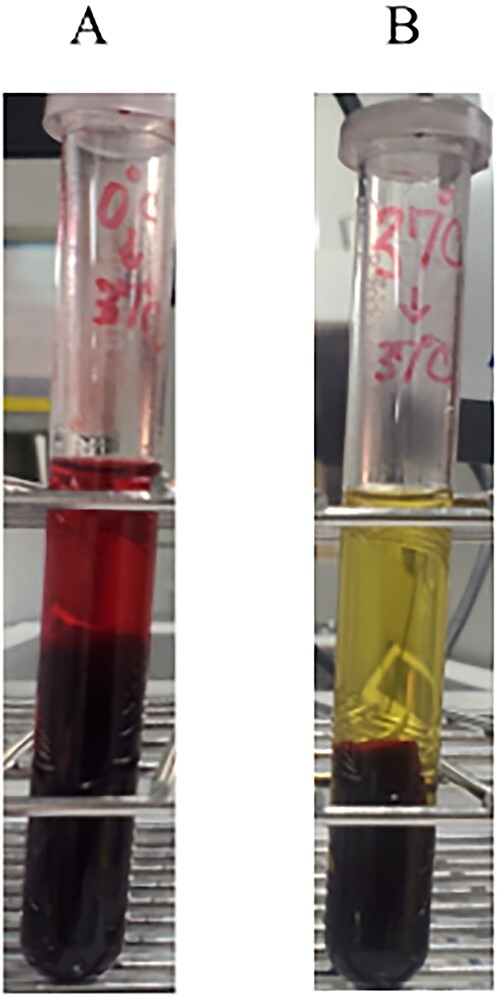
Donath-Landsteiner test. (**A**) 0°C for 30 min, followed by 37°C for 30 min. (**B**) 37°C for 60 min (control).

The D-L test results were positive and the cold agglutination titer was 64× (Fig. 2). These results confirmed the PCH diagnosis. After an additional antibiotic treatment for the latent syphilis (doxycycline 200 mg/day) along with avoiding any exposure to cold by keeping himself warm, the hemolytic anemia improved. The antibiotics were changed to doxycycline 200 mg/day for oral switch. The patient’s progress after discharge from the hospital is unknown because he transferred to another hospital.

## DISCUSSION

PCH is one AIHA in which complement activation by D-L antibodies causes intravascular hemolysis. In 1904, Donath and Landsteiner demonstrated the presence of causative autoantibodies and established the original concept for this disease [2]. Although PCH used to be frequently seen in syphilitic cases, presently it is primarily seen in children and often develops after viral infections and other diseases [3].

In the current case, hemolytic anemia was suspected based on blood tests and clinical symptoms. As a direct Coombs test was positive, this was suggestive of a diagnosis of AIHA. Urinalysis demonstrated there was a decreased haptoglobin, which is a finding of hemoglobinuria. A CT scan indicated that there was no splenomegaly, which suggested intravascular hemolysis. Previous findings have indicated that warm AIHA is unlikely to be the cause of intravascular hemolysis, as warm AIHA is predominantly associated with extravascular hemolysis [4]. Although a bone marrow biopsy was negative for malignant disease in this current case, the possibility of remained Paroxysmal nocturnal hematuria (PNH). With regard to the clinical course of the patient, improvement in the hemolytic symptoms that occurred without direct treatment for AIHA after his admission suggested that PNH was not likely [5]. Cold agglutinin disease (CAD) and PCH are diseases that cause intravascular hemolysis. This patient had repeated episodes of paroxysmal intravascular hemolysis during the winter season when he was admitted, and thus, CAD and PCH also came up in the differential diagnosis. A D-L test was performed in order to further evaluate the patient for PCH, with the positive results ultimately leading to the diagnosis of PCH.

The treatment of PCH is basically supportive therapy, such as avoiding cold exposure, keeping the patient warm, and the use of steroids, which have been reported to be effective and may be considered for use in severe cases [6]. Although reports of syphilitic PCH have been extremely rare in recent years, it has been reported that antibiotic treatment for syphilis may reduce or eliminate hemolysis. Although the efficacy of steroids in syphilitic PCH is not known, one study reported that steroids were effective during the acute phase of PCH in an elderly patient [7].

In our current case, the hemolysis improved in conjunction with avoidance of cold exposure by keeping the patient warm along with antibiotic treatment for the syphilis. Treatment with steroids was not considered as the patient improved soon after admission.

However, the disease recurred even after initiation of antibiotic treatment, so avoidance of cold exposure might have been more useful in this case.

Furthermore, it has been suggested that early intervention may prevent severe disease, and thus, it is important to take a history and perform appropriate tests in order to evaluate these types of patients.

When evaluating patients for PCH, which is a rare complication in syphilis-infected patients, consideration of cold exposure and testing for syphilis may be important factors in helping to differentiate from hemolytic anemia.
